# DHW-208, A Novel Phosphatidylinositol 3-Kinase (PI3K) Inhibitor, Has Anti-Hepatocellular Carcinoma Activity Through Promoting Apoptosis and Inhibiting Angiogenesis

**DOI:** 10.3389/fonc.2022.955729

**Published:** 2022-07-12

**Authors:** Shu Wang, Yuting Wu, Mingyue Liu, Qingchun Zhao, Lingyan Jian

**Affiliations:** ^1^ Department of Pharmacy, Shengjing Hospital of China Medical University, Shenyang, China; ^2^ Department of Clinical Pharmacy, Shenyang Pharmaceutical University, Shenyang, China; ^3^ Department of Pharmacy, China Medical University, Shenyang, China

**Keywords:** apoptosis, angiogenesis, proliferation, hepatocellular carcinoma, PI3K/AKT/mTOR pathway

## Abstract

Hepatocellular carcinoma (HCC) is one of the most common tumors worldwide with high prevalence and lethality. Due to insidious onset and lack of early symptoms, most HCC patients are diagnosed at advanced stages without adequate methods but systemic therapies. PI3K/AKT/mTOR signaling pathway plays a crucial role in the progression and development of HCC. Aberrant activation of PI3K/AKT/mTOR pathway is involved in diverse biological processes, including cell proliferation, apoptosis, migration, invasion and angiogenesis. Therefore, the development of PI3K-targeted inhibitors is of great significance for the treatment of HCC. DHW-208 is a novel 4-aminoquinazoline derivative pan-PI3K inhibitor. This study aimed to assess the therapeutic efficacy of DHW-208 in HCC and investigate its underlying mechanism. DHW-208 could inhibit the proliferation, migration, invasion and angiogenesis of HCC through the PI3K/AKT/mTOR signaling pathway *in vitro*. Consistent with the *in vitro* results, *in vivo* studies demonstrated that DHW-208 elicits an antitumor effect by inhibiting the PI3K/AKT/mTOR-signaling pathway with a high degree of safety in HCC. Therefore, DHW-208 is a candidate compound to be developed as a small molecule PI3K inhibitor for the treatment of HCC, and our study provides a certain theoretical basis for the treatment of HCC and the development of PI3K inhibitors.

## Instruction

Hepatocellular carcinoma (HCC) is one of the most common malignant tumors in the digestive tract (1). Chemotherapy is currently the main treatment for HCC (2, 3). However, the overall survival of some HCC patients after chemotherapy is only prolonged by a few months due to tumor drug resistance, metastasis and recurrence (4, 5). Therefore, curing HCC remains a challenge in the medical community (6, 7).

In the past few years, immunotherapy has made some progress in treating cancer, and Atezolizumab/Bevacizumab is the first and only immunotherapy with a proven benefit in HCC (8–10). However, the treatment options for advanced HCC are still very limited (11, 12). Sorafenib is a multikinase inhibitor capable of facilitating apoptosis, mitigating angiogenesis and suppressing tumor cell proliferation and remains the representative approved systemic treatment for advanced HCC (13, 14). The PI3K/AKT/mTOR signaling pathway plays an important role in physiological processes and is related to cell growth, survival and other processes (15–17). Abnormal activation of the PI3K/AKT/mTOR signaling pathway has been found in a number of cancers affects nearly 50% of malignant tumors, including HCC, and mediates cancer cell proliferation, migration, invasion, angiogenesis and other pathological processes in tumor cells (18–20). Compared with traditional chemotherapy drugs, molecular-targeted drugs have the advantages of clear targeting, enhanced efficacy and low toxicity (21, 22). In recent years, the role of targeted RTK drugs in the treatment of HCC has attracted much attention (23, 24). In response to growth factors or cytokines, RTK recruits PI3K to the cell membrane and mediates a cascade of downstream reactions through direct or indirect activation of the PI3K/AKT/mTOR pathway (25, 26). Currently, there are five FDA-approved PI3K inhibitors and there are many PI3K inhibitors are in clinical studies. But no PI3K inhibitors for HCC are currently on the market (27, 28).

The PI3K/AKT/mTOR signaling pathway also regulates multiple biological functions in HCC. One of the important pathological mechanisms of HCC disease progression is abnormal activation of the PI3K/AKT/mTOR signaling pathway (15, 29). Therefore, targeting PI3K to inhibit the PI3K/AKT/mTOR signaling pathway and its downstream effector molecules may be critical for HCC treatment (30). The development of targeted inhibitors of the PI3K/AKT/mTOR signaling pathway in HCC is very important in understanding the pathological mechanism of HCC (31–33). The sequential phosphorylation of PI3K, AKT and mTOR mediates the activation of a series of related molecular pathways and participates in the regulation of various biological functions. In apoptosis, AKT-mediated phosphorylation of proapoptotic proteins inhibits their activation, and AKT promotes the phosphorylation of the Bcl-2 family Bax at Ser184, which regulates the proapoptotic effect of Bax (34, 35). In cell metastasis, AKT can promote the transcriptional activation of TGF-β-mediated EMT. Moreover, mTOR can act on the transcription factor slug and inhibit its expression by binding to the promoter of E-cadherin (36–38). Slug also promotes the expression of MMP-9 and MMP-2 to activate EMT (39). In angiogenesis, mTOR mediates its effects by phosphorylating 4E-BP1 and disrupting the integrity of the complex formed by mTOR and 4E-BP1, which is critical for inhibiting the translation of related genes and enhances HIF-1α translation (40, 41). HIF-1α promotes transcriptional activation of VEGF, which stimulates neovascularization (42). Therefore, focusing on the PI3K/AKT/mTOR signaling pathway is crucial for the treatment of HCC.

**Figure 1:**
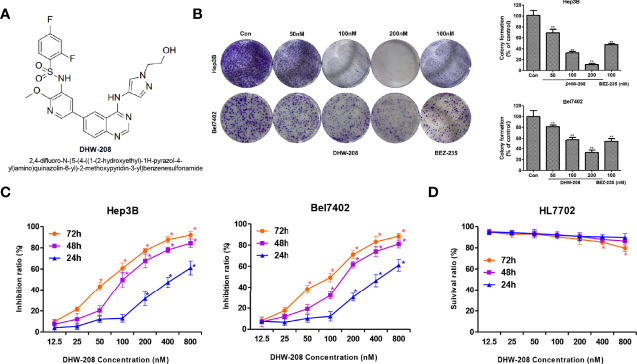
The proliferation inhibitory effect of DHW-208 on Hep3B, Bel7402 and HL7702 cell lines. **(A)** Structure of DHW-208. **(B)** Effect of DHW-208 on Hep3B, Bel7402 cell proliferation as evaluated with the colony formation assay. Bar graphs of the quantitative results were shown right. **(C)** MTT assay of Hep3B, Bel7402 cells treated with DHW-208 for 24, 48, and 72 h. **(D)** MTT assay of HL7702 cells treated with DHW-208 for 24, 48, and 72 h. Each value is the mean (± SD) from triplicate samples. *p < 0.05, **p < 0.01 *vs*. control.

In recent years, 4-amino-quinazoline derivatives, which are kinase inhibitors have attracted attention in the field of pharmaceutical chemistry (43, 44). These 4-amino-quinazoline derivatives have also been reported to be inhibitors of PI3Kα and PI3Kδ, suggesting that 4-amino-quinazoline derivatives are potential targeted antitumor drugs (6, 22, 45). DHW-208, a pan PI3K inhibitor, is a novel 4-amino-quinazoline derivative containing hydrophilic groups and suppressed the growth of cancer cells by inhibiting the PI3K/AKT/mTOR-signaling pathway (**Figure 1A**). Further research found that DHW-208 showed excellent inhibitory effects on HCC (45) which was a promising candidate for the treatment of HCC. This study aimed to explore the mechanism by which DHW-208 inhibited HCC, and lay a foundation for the development of targeted drugs for the treatment of HCC.

## Methods and Materials

DHW-208 was synthesized by Pharmaceutical chemistry laboratory, Shenyang Pharmaceutical University, Shenyang, China. Sorafenib was purchased from Solebo Co., LTD (Beijing, China). For the cellular experiments, DMSO (dimethyl sulfoxide) was used to dissolve the pure DHW-208 powder (also the other agents), and then use DMEM (Logan, UT, USA) without FBS to dilute the DHW-208 (also the other agents) DMSO-solution to prepare solutions containing a series of concentrations DHW-208. For the animal experiments, DMSO was used to dissolve the DHW-208 powder, and then PEG400, Tween 80 and saline (DMSO: Tween 80:PEG400:saline = 1:5:60:34) was used to dilute DHW-208 solutions by a series of concentrations.

### Cell Culture

The Hepatocellular carcinoma cell lines, Hep3B, Bel7402, HepG2, LM3 and MHCC97H, and hepatocyte HL7702 were purchased from American Type Culture Collection (ATCC, Manassas, VA, USA). All cells were cultured in DMEM supplemented with 10% fetal bovine serum (FBS) and incubated in an environment at 37°C containing 5% CO2.

### Antiproliferative Activity

Cell viability was assessed with MTT assay. Cells were seeded in 96-well plates in complete medium. After being incubated overnight they were exposed to diverse concentrations of DHW-208 for 24 h, 48 h and 72 h. The cells were then analyzed using the MTT (0.5 mg/ml) assay and measured with microplate-reader (Elx 800 Bio-Tek, USA).

### Colony Formation Assay

Hep3B and Bel7402 cells (1×10^3^ cells/well) were seeded into six-well plates, cultured overnight, and treated with DMSO or DHW-208 at different concentrations for 72 h. Then washed with PBS and cultured in full growth medium for another 7 days. The fresh medium was replaced every 3 days. After fixed with 100% methanol, the cells were stained with 0.1% crystal violet and quantified after being dissolved with glacial acetic acid. The plates were then analyzed with a microplate reader.

### Cell Morphology Analysis

Hep3B and Bel7402 cells were treated with DHW-208 for 48 h, then stained with Hoechst 33342 (Beyotime, Shanghai, China). After washed with PBS twice, the samples were photographed under fluorescence microscope (Olympus, Japan).

### Annexin FITC/PI Assay

After treated with DHW-208 for 48h, cells were fixed with 70% ethanol overnight, and stained with Annexin-V FITC/PI for 30 min in the dark before tested by fluorescence-activated cell sorting (FACS) (Becton-Dickinson, NJ, USA). Data was analyzed with Flow Jo.7.6.1 (Tree Star, Ashland, OR, USA).

### Transmission Electron Microscopy

Cells were collected and fixed with 3% glutaraldehyde. Then the samples were postfixed with 1% OsO4, then dehydrated in ascending series of ethanol, embedded, and sectioned. Stained with uranyl acetate and lead citrate, the samples were observed under an H-7650 transmission electron microscope (Hitachi, Japan).

### Western Blot Analysis

RIPA buffers, including protease inhibitors, homogenized cells and tumor tissues. Protein concentrations were determined by BCA protein detection kit. The proteins were separated by SDS–PAGE and transferred to PVDF membrane by electrophoresis. Membranes were immunoblotted using specific primary antibodies and then incubated the membrane with HRP-conjugated secondary antibody. The immune response bands were observed with the ECL assay kit. Blots were imaged by Image Quant LAS 4000 (GE Healthcare Life Sciences, Piscataway, NJ, USA).

### Wound Healing Scratch Assay

Hep3B (3×10^5^/ml) and Bel7402 cells (5×10^5^/ml) were seeded into 6-well plates. Confluent cells were scraped across the diameter of the well with a 200-mL pipette tip. The migration ability of the cells was tested after DHW-208 treatment for 48 h. Then cells were washed with PBS twice. The migration distance was photographed under microscope (Olympus, Japan). Image J software was used to determine the wound area.

### Migration, Invasion and Co-Culture Assay

Cells invasion assay was measured with 24-well transwell plate (Corning Life Sciences, MA, USA). Cells in serum-free medium were seeded onto the upper chamber uncoated or coated with Matrigel (Becton Dickinson, CA, USA). The lower chamber was filled the complete medium containing 10% FBS. In the co-culture experiment, another cell was added or not added to the lower chamber. After 48 h, the remaining cells on the upper side of the membrane were wiped with cotton swabs. The bottom side were fixed with 4% paraformaldehyde. The cells were stained with 0.1% crystal violet and counted under a microscope (Olympus, Japan).

### The *In Vivo* Anti-Tumor Activation of DHW-208 *via* a Nude Mice Model

All animal studies were obtained from Beijing Vital River Laboratory Animal Technology in accordance with the guidelines of the Animal Experimental Ethics Committee of Shengjing Hospital of China Medical University and complied with the internationally recognized Animal Research: Reporting of *In vivo* Experiments guideline. Hep3B cells were cultured and injected into the nude mice’s subcutaneous tumor position (5×10^6^ for each nude mice). The mice were randomized into five groups (n = 8) that administered with 0.2 mL vehicle, Sorafenib (10 mg/kg), and DHW-208 (10, 20, and 40 mg/kg) by oral gavage daily for 14 days. At the end of the experiment, all tumors and organs were removed and measured. The inhibitory rates of DHW-208 on Hep3B cells’ subcutaneous growth was calculated according to the tumor volumes or tumor weights.

### Hematoxylin and Eosin (H&E) Staining

After the nude mice were sacrificed, an autopsy was performed, and the main organs were removed. The samples were fixed in 10% neutral buffered formalin. Embedded in paraffin, the tumor samples were cut into 5 μm thickness and stained with H&E. Finally, the tumor tissues were observed under a microscope (Olympus, Japan).

## Results

### DHW-208 Inhibits HCC Cell Proliferation

First, the inhibitory effects of DHW-208 on the proliferation of Hep3B, Bel7402, HepG2, LM3 and MHCC97H HCC cells were investigated. The MTT results showed that after 72 h of treatment with different concentrations of DHW-208, the growth of HCC cells, especially Hep3B and Bel7402 cells, was significantly inhibited (**Table 1**). The colony formation assay also showed that DHW-208 inhibited the growth of Hep3B and Bel7402 cells in a concentration-dependent manner (**Figure 1B**). DHW-208 inhibited the growth of Hep3B and Bel7402 cells in concentration- and time-dependent manners, respectively (**Figure 1C**). The IC_50_ values of Hep3B cells at 24 h, 48 h and 72 h were 313.61 ± 1.48 nM, 92.85 ± 3.85 nM and 60.68 ± 0.85 nM, respectively. Moreover, the IC_50_ values of Bel7402 cells at 24 h, 48 h and 72 h were 360.20 ± 4.72 nM, 177.63 ± 1.13 nM and 80.31 ± 1.03 nM, respectively. Next, the cytotoxicity of DHW-208 to human hepatocytes was investigated. The effects of different concentrations of DHW-208 on the growth of normal human hepatocytes (HL7702) at 24 h, 48 h and 72 h were analyzed. The results showed that DHW-208 induced no significant toxicity in HL7702 cells for 24 h, 48 h and 72 h, suggesting that DHW-208 may have relatively low cytotoxicity (**Figure 1D**).

**Table 1:** The proliferation inhibitory effect of DHW-208 on HCC cells at 72 h.

HCC cell lines	IC_50_ (nM) of 72 h
Hep3B	60.68 ± 0.85
Bel7402	80.31 ± 1.03
MHCC97H	277.19 ± 2.95
LM3	584.15 ± 19.72
HepG2	755.79 ± 17.95
HL7702	2355.86 ± 33.86

### DHW-208 Inhibits HCC Tumor Growth *In Vivo*

**Figure 2:**
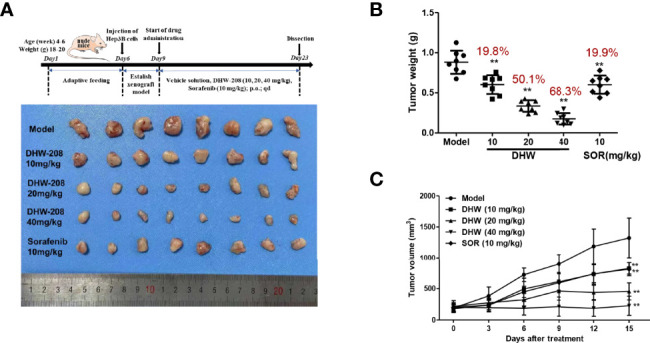
DHW-208 induces a potent antitumor effect in Hep3B nude mouse xenograft model. Hep3B was cultured and injected in to the subcutaneous position of the nude mice. Mice were received the DHW-208 and Sorafenib *via* oral administration. **(A)** Images of resected HCC tumor samples. **(B)** Average tumor weight at the end of the indicated treatment. **(C)** Average tumor volumes were measured every 3 days. Data are shown as mean ± SD (n=8). *p < 0.05, **p < 0.01 *vs*. Model.

To investigate the inhibitory effect of DHW-208 on HCC cell proliferation *in vivo*, a BALB/C xenograft tumor model bearing Hep3B cells was used. To evaluate the *in vivo* effects of DHW-208, Sorafenib, a first-line treatment for HCC, was selected as a positive control drug. **Figure 2A** shows a schematic diagram of the tumor morphology in each experimental group after drug administration. **Figures 2B, C** shows the statistical analysis of tumor weight and tumor volume in each group. The results showed that DHW-208 (10, 20 and 40 mg/kg) inhibited tumor growth in nude mice compared with mice in the model group, and the tumor weight in the high, medium and low concentration groups was significantly lower than that in the model group. Compared with those in the model group and DHW-208 (10, 20, 40 mg/kg) groups, the tumor inhibition rates were 19.8%, 50.1%, and 68.3%, respectively, and the tumor inhibition rate induced by 10 mg/kg Sorafenib was 19.9%.

**Figure 3:**
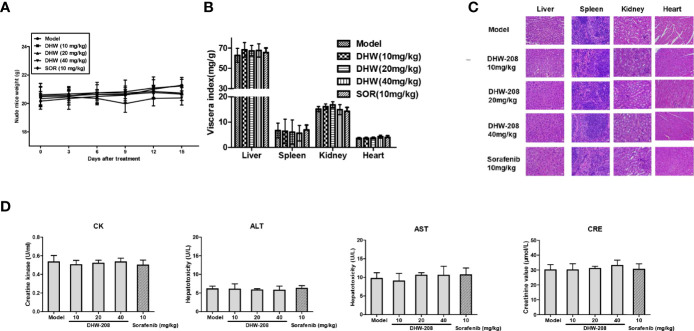
DHW-208 has no obvious effect on body weight change and organ toxicity in tumor-bearing nude mice. **(A)** Body weight change curve of nude mice after DHW-208 and Sorafenib administrations. **(B)** The viscera index of organs (heart, liver, spleen, lung and kidney). **(C)** HE staining of the heart, liver, kidney, and spleen from orthotopic nude mice. Scale bar = 100 µm. **(D)** The biochemical parameters of heart (CK), liver (ALT, AST) and kidney (CRE) from the serum of each group in tumor-bearing nude mice. Data are shown as mean ± SD (n=8). *p < 0.05, **p < 0.01 *vs*. Model.

During the experiment, the mental and activity states of nude mice were observed each day, and no abnormal conditions were found. The body weights of nude mice were recorded every three days, as shown in **Figure 3A**. No significant toxicity or body weight change was observed in response to DHW-208 at concentrations of 10 mg/kg, 20 mg/kg, or 40 mg/kg. Visceral index statistics showed that there were no significant differences in visceral indices of the heart, liver, kidney and spleen between the DHW-208 treatment group, the model group or the positive control group (**Figure 3B**). To further clarify the *in vivo* toxicity of DHW-208, we examined histological changes in the heart, liver, spleen and kidney by H&E staining. Compared with those in the model group, no obvious inflammatory infiltration or other histological abnormalities were observed in the liver, myocardium, glomerulus or splenic corpuscle in the DHW-208 treatment groups (**Figure 3C**). The effects of DHW-208 on the heart, liver and kidney were investigated by measuring biochemical indices in the orbital blood of nude mice. The results showed that there were no significant differences in CK, ALT, AST and CRE in the experimental DHW-208 groups compared with the model group (**Figure 3D**). These results indicated that DHW-208 could effectively inhibit tumor growth in nude mice without obvious visceral toxicity at the tested dose.

### DHW-208 Induces Apoptosis in HCC Cells

**Figure 4:**
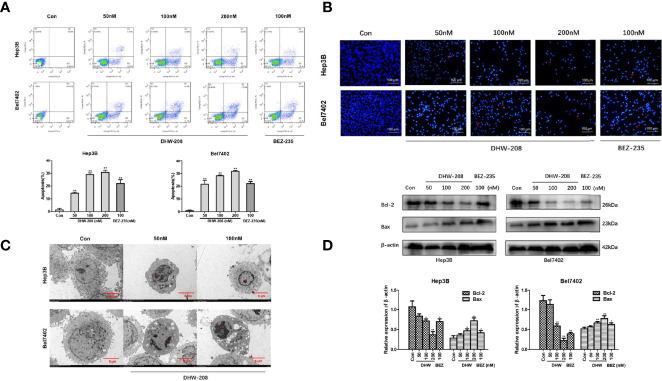
DHW-208 treatment causes cell apoptosis in HCC cells. **(A)** Annexin V-FITC/PI double-staining of cells treated with DHW-208 for 48h. The Annexin V-FITC/PI double-staining was quantified and plotted down. **(B)** Changes in cells treated with DHW-208 for 48h visualized by Hoechst 33342 staining (×200 magnification, scale bar = 100 μm). Arrows, apoptotic cells. **(C)** Morphologic changes in cells treated with DHW-208 were observed after 48h by transmission electron microscopy (×2500 magnification, scale bar = 5 μm). Red arrow, typical apoptotic micronuclei. **(D)** Changes in Bcl-2 and Bax in cells treated with DHW-208 48h by western blot. Bar graphs of the quantitative results were shown down. Each value is the mean ( ± SD) from triplicate samples. *p < 0.05, **p < 0.01 *vs*. control.

First, the effect of DHW-208 on Hep3B and Bel7402 cell apoptosis was investigated. **Figure 4A** shows that DHW-208 treatment for 48 h significantly increased the proportion of apoptotic Hep3B and Bel7402 cells in a concentration-dependent manner, regardless of whether they were early or late apoptotic cells. Then, the proapoptotic effect of DHW-208 was confirmed by Hoechst 33342 staining. As shown in **Figure 4B**, after Hoechst 33342 staining, HCC cells were generally light blue. After DHW-208 treatment for 48 h, the number of cells decreased in a concentration-dependent manner, and the proportion of bright blue cells increased. After apoptosis, the HCC cells underwent nuclear fragmentation and chromatin shrinkage. Transmission electron microscopy showed that HCC cells exhibited typical apoptotic characteristics, including chromatin condensation and margination at the nuclear periphery after DHW-208 treatment for 48 h. As shown in **Figure 4C**, Hep3B control cells had clear spacing and intercellular connections, but the connections were not tight. Hep3B cells that were treated with DHW-208 showed increased heterochromatin in the nucleus and had condensed into apoptotic bodies. Similar results were observed in Bel7402 cells (**Figure 4C**). Then, we further investigated the effect of DHW-208 on the BCL-2 family, which are key proteins of the endogenous apoptosis pathway, by Western blotting (**Figure 4D**). The results showed that DHW-208 increased the level of the representative proapoptotic protein Bax and decreased the level of the representative antiapoptotic protein Bcl-2 in a concentration-dependent manner.

### DHW-208 Inhibits Migration, Invasion in HCC Cells

**Figure 5:**
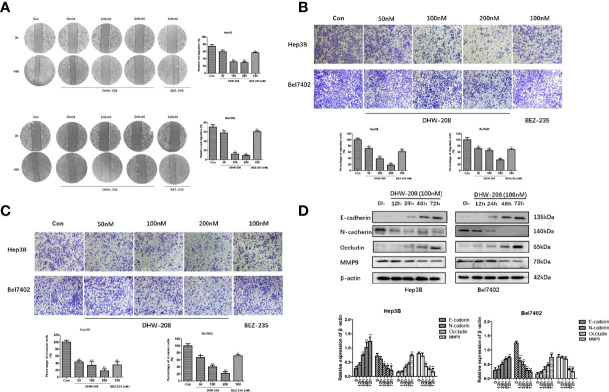
Effects of DHW-208 on HCC cells migration, invasion and EMT. **(A)** The effect of DHW-208 on cell migration of Hep3B and Bel7402 cells was measured by wound healing assay (×100 magnification). The migration rates were calculated by the formula shown right. **(B)** Transwell assay was performed to assess the migration of Hep3B and Bel7402 cells (×200 magnification). Bar graphs showed the quantitative results of the migration (down). **(C)** Transwell assay was performed to assess the invasion of Hep3B and Bel7402 cells (×200 magnification). Bar graphs showed the quantitative results of the invasion (down). **(D)** Western blot for the levels of EMT-related proteins (E-cadherin, N-cadherin, Occludin and MMP9) in Hep3B and Bel7402 cells treated with DHW-208 (100nM) for 0-72 h. Bar graphs of the quantitative results were shown down. Each value is the mean ( ± SD) from triplicate samples. *p < 0.05, **p < 0.01 *vs*. control.

Studies have shown that the PI3K pathway is involved in the migration, invasion and EMT of HCC cells (39, 46). The effects of different concentrations of DHW-208 on the migration and invasion of Hep3B and Bel7402 cells were investigated. Hep3B and Bel7402 cells were treated with DHW-208, scratch and invasion assays were performed, and the cells were photographed at 0 h and 48 h, respectively (**Figure 5A**). The results showed that compared with those in the control group, the migration and invasion of HCC cells in the DHW-208 treatment group were significantly reduced, and the effects on migration and invasion were concentration-dependent (**Figures 5B, C**). The results of the scratch and invasion assays indicated that DHW-208 could effectively inhibit the migration and invasion of HCC cells. Next, the effects of DHW-208 on EMT-related protein expression were investigated. As shown in **Figure 5D**, DHW-208 significantly increased the expression of epithelial cell markers (E-cadherin and Occludin) and downregulated the expression mesenchymal cell markers (N-cadherin) in Hep3B and Bel7402 cells in a concentration-dependent manner. In addition, matrix metalloproteinase (MMP) can degrade various protein components in extracellular matrix, accelerate the hydrolysis of intercellular adhesion proteins, and promote EMT of tumor cells as the first barrier during tumor metastasis. Therefore, activation of MMPS indirectly mediates tumor migration, invasion and angiogenesis. DHW-208 also significantly inhibited the expression of the tumor metastasis marker MMP9 in Hep3B and Bel7402 cells in a concentration-dependent manner.

### DHW-208 Inhibits Angiogenesis in HCC Cells

**Figure 6:**
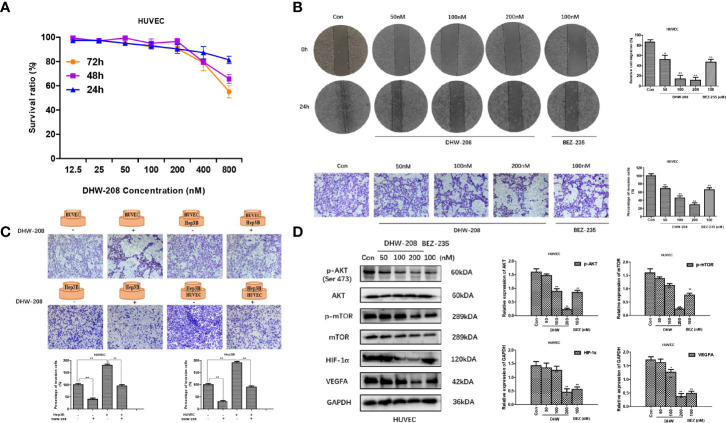
The effect of DHW-208 on cell migration, invasion and angiogenesis in HUVECs. **(A)** The effect of DHW-208 on cell survival in HUVECs by MTT assay. **(B)** The effect of DHW-208 on cell migration and invasion in HUVECs (×100 magnification). Bar graphs showed the quantitative results of the migration and invasion (right). **(C)** The role of DHW-208 in cell interaction between Hep3B cells and HUVECs by cell co-culture assay (×200 magnification). Bar graphs showed the quantitative results of the migration and invasion (down). **(D)** DHW-208 treatment for 48h observably reduced the expression level of proangiogenic proteins (VEGFA and HIF-1α) and PI3K pathway-related proteins (p-AKTser473 and p-mTOR). Bar graphs of the quantitative results were shown right. Each value is the mean (± SD) from triplicate samples. *p < 0.05, **p < 0.01 *vs*. control.

Rapid tumor proliferation could lead to ischemia and hypoxia, which produced pro-angiogenic factors and hypoxia-inducible factors, and further promoted the proliferation and migration of endothelial cells and the formation of new tumor blood vessels. These factors aggravate the tumor deterioration. HUVECs are human umbilical vein endothelial cells that are often used *in vitro* to examine angiogenesis (47). The results showed that DHW-208 had low toxicity to HUVECs (**Figure 6A**). The effects of DHW-208 on the migration and invasion of HUVECs were investigated. Cell scratch and transwell invasion assays showed that DHW-208 could significantly inhibit the migration and invasion of HUVECs (**Figure 6B**). These results suggest that DHW-208 can significantly inhibit the metastasis of HUVECs at the tested concentrations without obvious toxicity to vascular endothelial cells, which may further affect the angiogenesis of HUVECs. Furthermore, we investigated the interaction between HUVECs and Hep3B cells by transwell coculture *in vitro*, which simulates the interaction between tumor cells and endothelial cells during angiogenesis and examines the role of the tumor microenvironment. The interaction between HUVECs and Hep3B cells was investigated by transwell assays. The results showed that HUVECs and Hep3B cells could interact with each other to mutually promote invasion, and DHW-208 could significantly inhibit the increase in invasion. These results showed that HUVECs and Hep3B cells could promote mutual invasion, while DHW-208 could significantly inhibit the increase in invasion (**Figure 6C**).

To further verify the antiangiogenic mechanism of DHW -208, we investigated whether DHW-208 could inhibit the PI3K signaling pathway in HUVECs. The Western blot results suggested that the protein expression levels of p-AKT (Ser473) and p-mTOR were decreased, while the levels of AKT and mTOR were not affected. DHW-208 also significantly inhibited the expression of the angiogenic factors VEGFA and HIF-1α (**Figure 6D**). These results suggest that DHW-208 can exert an antiangiogenic effect through the PI3K pathway.

## Discussion

The PI3K/AKT/mTOR signaling pathway is involved in a variety of pathological mechanisms, including proliferation, apoptosis, metastasis, invasion, and angiogenesis (40, 48). Deregulation of the PI3K/Akt/mTOR pathway leading to activation is common in HCC and is hence the subject of intense investigation and the focus of current therapeutics. Because this pathway is important in the pathological mechanism of HCC, the development of small molecule inhibitors targeting PI3K has attracted much attention (49–51). Currently many PI3K inhibitors as anti-tumor drugs have been developed, but only five PI3K inhibitors were approved by FDA. And there are still no marketed PI3K inhibitors for HCC. Based on the current research progress of PI3K inhibitors, the anti-hepatocellular carcinoma activity of the novel 4-amino-quinazoline derivative DHW-208 was explored in our study. DHW-208 inhibited the proliferation of HCC both *in vitro* and *in vivo*, and the mechanism was further studied. This study provides ideas and a theoretical basis for the development of PI3K inhibitors for the treatment of HCC.

HCC is characterized by abnormal cell proliferation caused by abnormal regulatory signals and proteins (52). Apoptosis is one of the main modes of cell death and is characterized by a series of changes in cell morphology and related regulatory enzymes (53). The Bcl-2 family is a key regulator of endogenous apoptosis, which is the main mechanism of apoptosis (54). Apoptosis can be triggered when the regulation of proapoptotic proteins exceeds that of antiapoptotic proteins (55). Studies have shown that this process is one of the earliest events in the apoptosis cascade and occurs before the changes in the nucleus (chromatin concentration, DNA fragmentation), and once this change occurs, cell apoptosis is irreversible (56). The PI3K/AKT/mTOR signaling pathway plays an important role in apoptosis (57). We found that DHW-208 could significantly inhibit the proliferation of Hep3B and Bel7402 HCC cells and promote HCC cell apoptosis by inducing the endogenous apoptosis pathway.

The abnormal activation of the PI3K pathway is also closely related to migration and invasion in HCC. EMT enhances the migration and invasion of HCC cells (58, 59). After EMT, HCC cells lose their epithelial-like morphology, downregulate epithelial marker expression, and reduce intercellular adhesion (60, 61). Then, HCC cells acquire mesenchymal cell morphology and upregulate the expression of mesenchymal markers, making them more prone to migration and invasion (61, 62). Angiogenesis occurs frequently in tumors. When the tumor proliferates rapidly, ischemia and hypoxia occur, and angiogenic factors and hypoxia-inducible factors are produced to promote the proliferation and migration of endothelial cells, resulting in the formation of new tumor blood vessels and exacerbating tumor deterioration (63, 64). Therefore, inhibiting endothelial cell migration, invasion and angiogenesis is crucial in controlling tumor progression.

Antiangiogenic drugs can effectively inhibit the growth, diffusion and metastasis of the primary tumor. We investigated the inhibitory effect of DHW-208 on the metastasis of HCC cells and found that DHW-208 could significantly inhibit migration and invasion in Hep3B and Bel7402 cells. DHW-208 effectively inhibited the migration and invasion of HUVECs, as shown by the cell scratch assay and transwell invasion assay. Fast-growing malignant tumor cells require adequate nutrient and oxygen transport, so more blood vessels are needed to promote cell overproliferation. However, due to vascular leakage, tumor cells can easily invade new blood vessels and form distant metastases without having to go through a complex process (14, 65). Therefore, it is necessary to investigate the interaction between tumor cells and endothelial cells for angiogenesis. *In vitro* transwell coculture experiments with HUVECs and Hep3B cells verified that DHW-208 could significantly inhibit the interaction between HUVECs and Hep3B cells. The formation of blood vessels is the result of the coordination between angiogenic factors and angiogenic inhibitors (66). These factors are in dynamic balance under normal condition, but once the balance is broken, excessive angiogenesis will occur (67, 68).

The upregulation of angiogenic factors can activate endothelial tyrosine kinases and downstream cascades *via* PI3K, mediating tumor angiogenesis (30). In the absence of a stable vascular system to provide adequate oxygen for growing tumors, the rapid proliferation of cancer cells leads to tumor hypoxia. Tumor hypoxia can increase the expression of HIF-1α, VEGF and other angiogenic factors. VEGFA is an angiogenic progenitor, and HIF-1α is an upstream factor of VEGFA and a key effector in the tumor microenvironment, both of which regulate angiogenesis (69, 70). PI3K signaling pathway plays a key role in tumor angiogenesis by regulating the expression of HIF-1α and VEGF. Activation of the PI3K/AKT/mTOR pathway in tumor cells can also increase VEGF secretion, both by hypoxia-inducible factor 1 dependent and independent mechanisms (11). Numerous inhibitors targeting the PI3K/AKT/mTOR pathway have been developed, and these agents have been shown to decrease VEGF secretion and angiogenesis (70). Hence, the PI3K pathway plays an important role in regulating angiogenesis in cancers. DHW-208 significantly inhibited the expression of p-AKT (Ser473) and mTOR, as well as the angiogenic factors VEGFA and HIF-1α, in HUVECs. These findings suggest that DHW-208 can play an antiangiogenic role through the PI3K pathway.

In conclusion, as a pan-PI3K inhibitor, DHW-208 can inhibit the proliferation, migration, invasion and angiogenesis of HCC by inhibiting the activation of PI3K/AKT/mTOR signaling pathway, exhibiting robust anti-HCC activity both *in vivo* and *in vitro*. DHW-208 is expected to be a potential selective small molecule PI3K inhibitor for the treatment of HCC, with certain potential for further development.

## Data Availability Statement

The original contributions presented in the study are included in the article/**Supplementary Materials**. Further inquiries can be directed to the corresponding authors.

## Ethics Statement

The animal study was reviewed and approved by The Animal Experimental Ethics Committee of Shengjing Hospital of China Medical University.

## Author Contributions

QZ, LJ, and SW conceived the main ideas and wrote the paper. YW supervised the study. SW, ML, and YW developed major methodologies, databases, reagents, and primary experiments. All authors contributed to the article and approved the submitted version.

## Funding

This research was supported by Postdoctoral Workstation of China Medical University.

## Conflict of Interest

The authors declare that the research was conducted in the absence of any commercial or financial relationships that could be construed as a potential conflict of interest.

## Publisher’s Note

All claims expressed in this article are solely those of the authors and do not necessarily represent those of their affiliated organizations, or those of the publisher, the editors and the reviewers. Any product that may be evaluated in this article, or claim that may be made by its manufacturer, is not guaranteed or endorsed by the publisher.
