# Supplementary material for: DHW-208, A Novel Phosphatidylinositol 3-Kinase (PI3K) Inhibitor, Has Anti-Hepatocellular Carcinoma Activity Through Promoting Apoptosis and Inhibiting Angiogenesis
Source: Front Oncol. 2022 Jul 12;12:955729. doi: 10.3389/fonc.2022.955729 (PMC9315107; doi:10.3389/fonc.2022.955729)
Supplement: Supplementary file 1 [file DataSheet_1.docx]

The download link of full original source data is located in <https://www.jianguoyun.com/p/DVVozr8Qt5jMChjUyMAEIAA>

, which have to set the sharing scope for the download link to “All registered users” because the sharing scope for the download link can not select from “Anyone, including non-registered users” If you have any questions regarding the download link, please do not hesitate to contact me.
